# Reverse Sequence Polymerization‐Induced Self‐Assembly in Aqueous Media

**DOI:** 10.1002/anie.202207376

**Published:** 2022-07-06

**Authors:** Thomas J. Neal, Nicholas J. W. Penfold, Steven P. Armes

**Affiliations:** ^1^ Department or Chemistry The University of Sheffield Brook Hill, Sheffield, South Yorkshire S3 7HF UK

**Keywords:** Block Copolymers, Charge-Stabilized Latex, Polymerization-Induced Self-Assembly, RAFT Aqueous Dispersion Polymerization

## Abstract

We report a new aqueous polymerization‐induced self‐assembly (PISA) formulation that enables the hydrophobic block to be prepared first when targeting diblock copolymer nano‐objects. This counter‐intuitive reverse sequence approach uses an ionic reversible addition–fragmentation chain transfer (RAFT) agent for the RAFT aqueous dispersion polymerization of 2‐hydroxypropyl methacrylate (HPMA) to produce charge‐stabilized latex particles. Chain extension using a water‐soluble methacrylic, acrylic or acrylamide comonomer then produces sterically stabilized diblock copolymer nanoparticles in an aqueous one‐pot formulation. In each case, the monomer diffuses into the PHPMA particles, which act as the locus for the polymerization. A remarkable change in morphology occurs as the ≈600 nm latex is converted into much smaller sterically stabilized diblock copolymer nanoparticles, which exhibit thermoresponsive behavior. Such reverse sequence PISA formulations enable the efficient synthesis of new functional diblock copolymer nanoparticles.

## Graphical abstract

Amphiphilic diblock copolymer nano‐objects are prepared in aqueous media using a highly counter‐intuitive formulation based on reverse sequence polymerization‐induced self‐assembly (PISA). The hydrophobic block is prepared first in the form of a ca. 600 nm charge‐stabilized latex using an ionic reversible addition–fragmentation chain transfer (RAFT) agent. Subsequent growth of a hydrophilic block produces sterically stabilized nanoparticles.

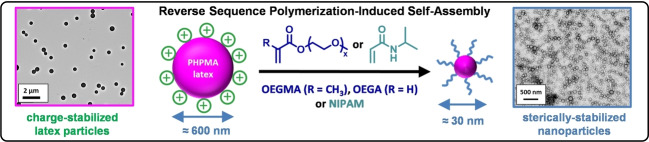

## Introduction

Block copolymer self‐assembly in solution to form sterically stabilized nanoparticles (also known as micelles) has been known for more than fifty years.[^1^, ^2^, ^3^, ^4^] Traditionally, such self‐assembly has been achieved using a post‐polymerization processing route.[^5^, ^6^, ^7^, ^8^, ^9^, ^10^, ^11^, ^12^] Typically, AB diblock copolymer chains are first dissolved in a good solvent for each block, then a non‐solvent for one of the two blocks is slowly added to induce nanoparticle formation.^[7]^ However, this “solvent switch” is invariably conducted in dilute solution, which has hitherto limited potential commercial applications. Over the past fifteen years or so, there has been growing interest in the preparation of block copolymer nanoparticles via polymerization‐induced self‐assembly (PISA).[^13^, ^14^, ^15^, ^16^, ^17^, ^18^, ^19^, ^20^, ^21^, ^22^, ^23^, ^24^] This involves chain extension of a soluble precursor block with a second block that gradually becomes insoluble as it grows in a poor solvent environment, thus driving self‐assembly during polymerization. In essence, the unreacted monomer acts as a processing aid (or co‐solvent) for in situ self‐assembly. This enables the convenient and efficient synthesis of many types of block copolymer nanoparticles (e.g. spheres, worms, rods, vesicles, lamellae, framboidal vesicles, inverse bicontinuous phases, etc.) in the form of concentrated dispersions.[^19^, ^25^, ^26^, ^27^, ^28^, ^29^, ^30^, ^31^, ^32^, ^33^, ^34^, ^35^, ^36^, ^37^, ^38^, ^39^] Moreover, judicious selection of appropriate monomer building blocks enables PISA to be conducted in a wide range of solvents, including water, polar solvents or non‐polar solvents.[^4^, ^17^, ^40^, ^41^, ^42^, ^43^] Most PISA syntheses involve reversible addition–fragmentation chain transfer (RAFT) polymerization,[^30^, ^44^, ^45^, ^46^, ^47^, ^48^, ^49^, ^50^, ^51^, ^52^, ^53^, ^54^, ^55^, ^56^, ^57^] although other living[^33^, ^58^, ^59^, ^60^] and pseudo‐living polymerization chemistries, such as iodine transfer polymerization,[^61^, ^62^] atom transfer radical polymerization (ATRP)[^63^, ^64^, ^65^, ^66^, ^67^] and nitroxide‐mediated polymerization (NMP)[^68^, ^69^, ^70^] are also applicable. A remarkably diverse range of potential applications for PISA‐synthesized nanoparticles have been suggested, including new wholly synthetic biocompatible 3D cell culture media,^[71]^ thermoresponsive hydrogels for long‐term storage of stem cells,^[72]^ enzyme encapsulation,[^73^, ^74^] bespoke flocculants for micron‐sized particles,^[75]^ organic opacifiers for paint formulations,[^25^, ^76^] new Pickering emulsifiers,[^36^, ^77^, ^78^] viscosity modifiers,[^79^, ^80^, ^81^] reinforcement additives for latex films,[^82^, ^83^, ^84^] ice recrystallization inhibitors,^[85]^ dispersants for agrochemical actives,[^86^, ^87^] and lubricating nanoparticles for automotive engine oils.^[88]^

**Scheme 1:**
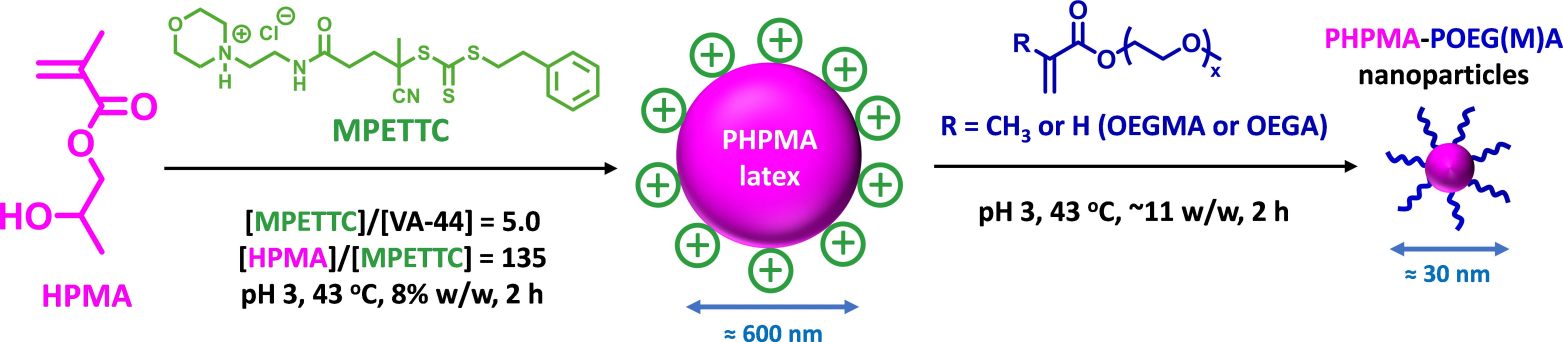
Representation of a one‐pot reverse sequence aqueous PISA formulation based on the RAFT aqueous dispersion polymerization of 2‐hydroxypropyl methacrylate (HPMA). The initial step is the formation of a cationic PHPMA_135_ latex, with this homopolymer precursor being subsequently chain‐extended using either oligo(ethylene glycol) methacrylate (OEGMA) or oligo(ethylene glycol) acrylate (OEGA) to form PHPMA_135_‐POEGMA_30_ or PHPMA_135_‐POEGA_20_ nanoparticles, respectively.

In the case of aqueous PISA formulations, it is seemingly axiomatic that the precursor block must be water‐soluble in order to confer steric stabilization on the final amphiphilic diblock copolymer nanoparticles.[^38^, ^39^, ^89^, ^90^, ^91^, ^92^, ^93^, ^94^] In the present study, we challenge this long‐standing paradigm by preparing the structure‐directing hydrophobic block first, followed by the hydrophilic stabilizer block. This is achieved by using a suitable water‐soluble ionic RAFT agent to prepare charge‐stabilized latex particles via surfactant‐free RAFT aqueous dispersion polymerization of 2‐hydroxypropyl methacrylate (HPMA). This PHPMA precursor is then chain‐extended to form the desired much smaller sterically stabilized diblock copolymer nanoparticles (see Scheme 1).

## Results and Discussion

In 2003 Shim et al.^[95]^ reported the RAFT aqueous emulsion photopolymerization of methyl methacrylate (MMA) using a surface‐active carboxylic acid‐based dithioester RAFT agent to prepare charge‐stabilized PMMA latex particles with mean diameters ranging from 304 to 407 nm. GPC analysis of such formulations indicated relatively high molecular weight PMMA chains (*M*_n_ up to 413 000, *M*_w_/*M*_n_≈1.30–1.40). Subsequently, Charleux and co‐workers reported using an anionic carboxylate‐functionalized RAFT agent for the surfactant‐free RAFT aqueous emulsion (co)polymerization of *n*‐butyl methacrylate with either *n*‐butyl acrylate or styrene at pH 8 to produce charge‐stabilized nanoparticles.^[96]^ Good colloidal stability was observed for the majority of the syntheses conducted in this prior study. However, a relatively high conversion (90 % after 213 min at 70 °C) and a reasonably low dispersity (*M*_w_/*M*_n_=1.36) was obtained in only one instance. This was achieved when *n*‐butyl methacrylate was statistically copolymerized with a small amount of *n*‐butyl acrylate to afford nanoparticles of 49 nm diameter. In contrast, only rather poor control and/or substantially incomplete conversions were obtained for the other seven syntheses.^[96]^ More recently, Moad and co‐workers^[97]^ demonstrated that RAFT emulsion polymerization of various less‐activated monomers (LAMs) and more‐activated monomers (MAMs) can be mediated using cationic dithiocarbamate‐based RAFT agents to afford charge‐stabilized nanoparticles with mean diameters of less than 100 nm. However, conventional surfactants were also utilized for such syntheses, presumably to ensure colloidal stability.

In 2007 we reported the preparation of PHPMA latexes via aqueous dispersion polymerization using conventional free radical chemistry and poly(*N*‐vinylpyrrolidone) (PNVP) as a steric stabilizer.^[98]^ The effect of initiator type, PNVP concentration and addition of surfactant on particle size was examined, with mean latex diameters of up to 1 μm being obtained. This study informed our early PISA syntheses, which involved the RAFT aqueous dispersion polymerization of HPMA using a water‐soluble precursor such as poly(glycerol monomethacrylate) as a steric stabilizer block.[^99^, ^100^, ^101^] Recently, we hypothesized that the surfactant‐free RAFT aqueous dispersion polymerization of HPMA should be feasible using solely charge stabilization, rather than steric stabilization. As far as we are aware, no other such formulations have been reported in the literature.

**Figure 1:**
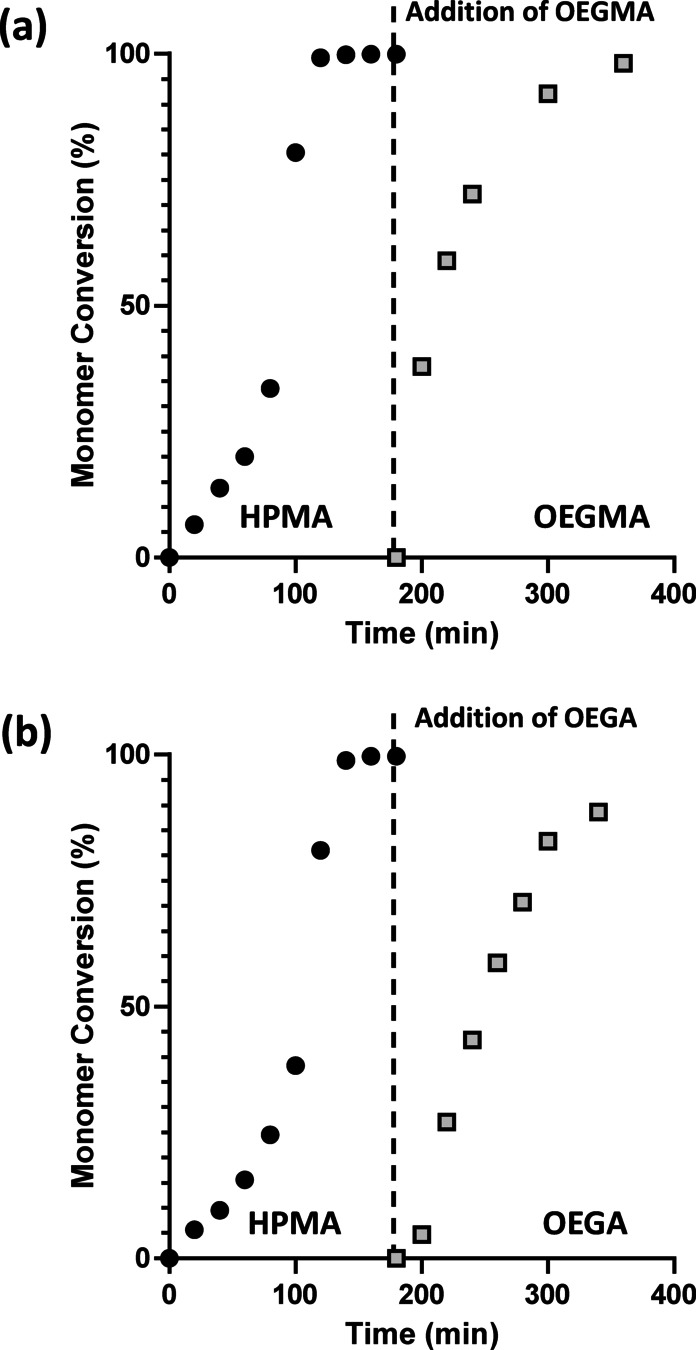
Monomer conversion vs. time curves obtained for the RAFT aqueous dispersion polymerization of HPMA using a cationic MPETTC RAFT agent at 43 °C, followed by extension of the PHPMA precursor chains using either a) OEGMA (target DP=30) or b) OEGA (target DP=20).

In the current work, a morpholine‐functionalized trithiocarbonate‐based RAFT agent (MPETTC) was synthesized as described previously.^[102]^ Protonation of its morpholine group ensures aqueous solubility at low pH, thus enabling the feasibility of a new surfactant‐free PISA formulation to be examined.^[102]^ Accordingly, the RAFT aqueous dispersion polymerization of HPMA was performed at pH 3 targeting 8 % w/w solids (Scheme 1). Kinetic data were obtained at 43 °C by periodic sampling of the reaction mixture followed by ^1^H‐NMR spectroscopy studies and GPC analysis (Figure 1a and S1a). In this particular experiment, the target DP for the PHPMA chains was 135. The conversion vs. time data indicate that a distinct change in the rate of polymerization occurs after 80 min, which corresponds to 34 % conversion or an instantaneous PHPMA DP of 46. This time point indicates micellar nucleation: a gradual increase in turbidity thereafter coincides with a dramatic increase in the rate of polymerization, with more than 99 % conversion being obtained within 2 h. Similar observations have been reported for conventional aqueous PISA syntheses and are attributed to monomer ingress within the nascent nanoparticles.[^90^, ^101^, ^103^] Visual inspection of the final latex dispersion indicated a milky‐white, free‐flowing fluid. Importantly, the linear evolution in *M*_n_ with HPMA conversion (Figure S2) and the relatively narrow molecular weight distributions (*M*_w_/*M*_n_ <1.10) confirm that this homopolymerization proceeds with excellent RAFT control.[^44^, ^50^, ^52^, ^55^, ^104^] Furthermore, DLS and aqueous electrophoresis analysis indicated that the final PHPMA latex had a z‐average diameter of 691 nm (DLS polydispersity=0.078) and a ξ‐potential of +57 mV at pH 3. Indeed, each PHPMA precursor latex prepared in this study exhibited a z‐average diameter of 576 to 691 nm. Moreover, very high final HPMA conversions were invariably achieved, thus demonstrating good reproducibility for the first step of this synthetic protocol (Table S1).

**Figure 2:**
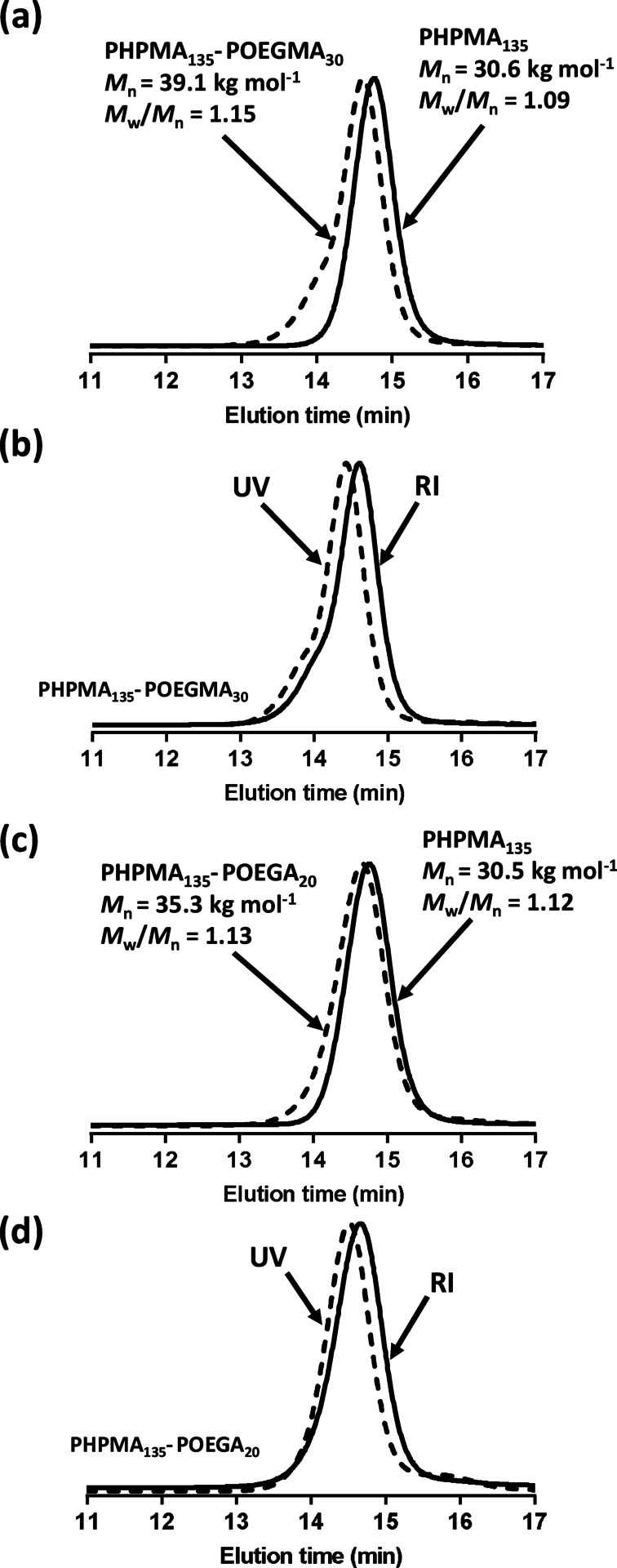
(a) DMF GPC curves recorded for the PHPMA_135_ latex precursor and the corresponding PHPMA_135_‐POEGMA_30_ nanoparticles using a refractive index detector. (b) Comparison between refractive index and UV detectors (*λ*=305 nm) for the same two samples. (c) DMF GPC curves recorded for the PHPMA_135_ latex precursor and the corresponding PHPMA_135_‐POEGA_20_ nanoparticles using a refractive index detector. (d) Comparison between refractive index and UV detectors (*λ*=305 nm) for the same two samples.

This initial PHPMA_135_ latex was then chain‐extended using either oligo(ethylene glycol) methyl ether methacrylate (OEGMA) or oligo(ethylene glycol) methyl ether acrylate (OEGA) to produce either PHPMA_135_‐OEGMA_30_ or PHPMA_135_‐POEGA_20_ diblock copolymer nanoparticles, respectively. [It is perhaps worth emphasizing here that the analogous POEGA_20_‐PHPMA_135_ nanoparticles cannot be prepared via conventional RAFT aqueous dispersion polymerization using a water‐soluble POEGA precursor owing to poor cross‐initiation efficiency when switching from an acrylic block to a methacrylic block.^[105]^] These reverse sequence PISA syntheses were performed using a highly convenient one‐pot protocol with either OEGMA or OEGA monomer being added to the PHPMA latex without any additional initiator (Scheme 1). Kinetic data were obtained for the second‐stage polymerization in each case (Figure 1 and S1). More than 90 % conversion was obtained within 2 h when using OEGMA, with 98 % conversion being achieved within 3 h. However, OEGA only reached 89 % monomer conversion within 3 h under the same conditions (a final monomer conversion of 91 % was achieved after 16 h). Both formulations produced a weakly turbid, yellow aqueous dispersion (with the color attributed to the trithiocarbonate RAFT groups). In each case, GPC analysis confirmed that the *M*_n_ increased on addition of the respective hydrophilic monomer (Figure 2 and S1).

Furthermore, the narrow molecular weight distribution observed for the precursor PHPMA block was retained after its chain extension (*M*_w_/*M*_n_<1.20). Importantly, the presence of trithiocarbonate groups on the copolymer chains was confirmed by GPC analysis using a UV detector set to 305 nm, which is the wavelength corresponding to the absorption maximum for these end‐groups. The UV and refractive index chromatograms are very similar for the PHPMA_135_‐OEGMA_30_ and PHPMA_135_‐POEGA_20_ formulations (Figure 2). In summary, GPC analysis confirms that excellent RAFT control is achieved during the synthesis of these two diblock copolymers and that no uncontrolled free radical homopolymerization of the OEGMA or OEGA occurred within the aqueous phase. This suggests that the locus of the second‐stage polymerization is solely within the PHPMA latex particles.

**Figure 3:**
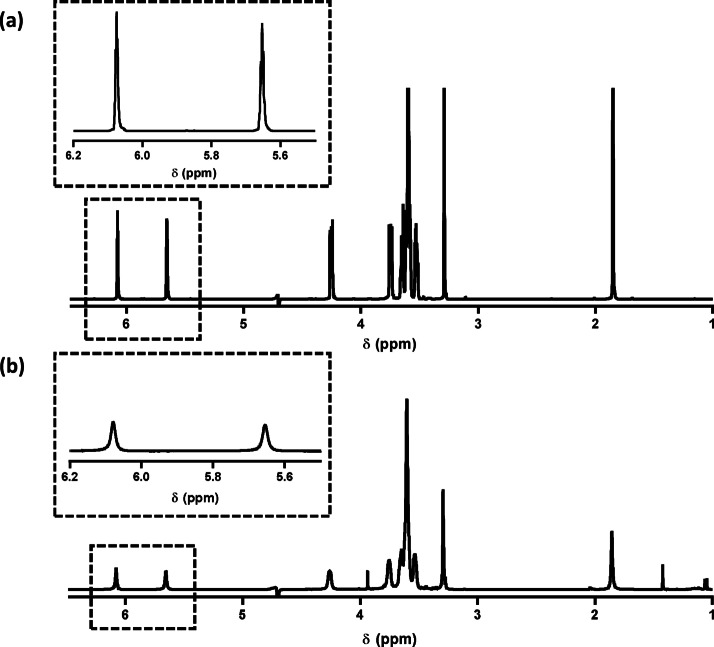
^1^H‐NMR spectra recorded for (a) OEGMA monomer (72 mg) dissolved in pH 3 water (2 mL plus 0.2 mL D_2_O) and (b) OEGMA monomer (72 mg) added to 2 mL of an 8 % w/w (plus 0.2 mL D_2_O) aqueous dispersion of PHPMA_135_ latex. Significant peak attenuation is observed for the OEGMA vinyl signals in the presence of the latex (compare insets), which suggests that this monomer preferentially locates within the latex particles rather than remaining in the aqueous phase.

To investigate monomer migration into the PHPMA latex, ^1^H‐NMR spectra were recorded for (i) OEGMA in weakly acidic aqueous solution (90 : 10 H2O/D2O, adjusted to pH 3 using HCl/DCl, see Figure 3a) and (ii) OEGMA in the presence of the 8 % w/w aqueous PHPMA latex under the same conditions (Figure 3b). Relatively narrow ^1^H‐NMR signals are observed for OEGMA monomer in aqueous solution, as expected for such a rapidly diffusing small molecule (Figure 3a). However, these signals become significantly more attenuated in the presence of the PHPMA latex particles (Figure 3b). This suggests that at least some of the OEGMA monomer migrates into the PHPMA latex. Similar NMR experiments were performed in the presence of an external standard (sodium benzenesulfonate) in order to quantify the proportion of OEGMA that migrates into the latex (Figure S3). Comparing the integral for the external standard against the integrated vinyl signal assigned to OEGMA in the absence and presence of the PHPMA latex particles, we estimate that the vinyl signal intensity is reduced by 62 % in the presence of the latex. These observations indicate that the OEGMA monomer migrates into the PHPMA latex and is hence able to react with the propagating polymer radicals buried within its interior.

We also attempted to chain‐extend a PHPMA_135_ latex using either glycerol monomethacrylate (GMA) or *N*,*N*‐dimethyl acrylamide (DMAC) to target PHPMA_135_‐PGMA_55_ and PHPMA_135_‐PDMAC_55_ nanoparticles, respectively. Interestingly, these reverse sequence PISA syntheses were unsuccessful. In striking contrast to the OEGMA and OEGA formulations described above, GPC analysis indicated broad high molecular weight shoulders when using a refractive index detector (Figure S4a–d). Moreover, UV GPC analysis confirmed that this feature contained no trithiocarbonate end‐groups. Thus, GMA and DMAC merely undergo uncontrolled free radical homopolymerization in the aqueous continuous phase, rather than growing from the PHPMA chains within the latex particles.

GMA, DMAC, OEGMA and OEGA are hydrophilic monomers with high aqueous solubility. In view of this, it is tempting to suggest that specific interactions such as hydrogen bonding might account for the stronger partitioning of OEGMA and OEGA into the PHPMA latex particles compared to that observed for GMA and DMAC. In both cases, the oligo(ethylene glycol) side‐chains should act as hydrogen bond acceptors for the hydrogen bond‐donating pendent hydroxyl groups on the PHPMA chains. However, this may be an overly simplistic interpretation, as evidenced by the findings presented below for a fourth water‐miscible monomer, *N*‐isopropylacrylamide (NIPAM).

When NIPAM is polymerized in the presence of a PHPMA_135_ latex, efficient chain extension of this hydrophobic precursor is observed when targeting a PNIPAM DP of 44 (Figure S4e, f). Importantly, there is no evidence for the uncontrolled free radical homopolymerization observed for the GMA and DMAC monomers. However, it is perhaps harder to explain why NIPAM monomer has significantly greater affinity for the PHPMA latex particles compared to GMA and DMAC monomers. Finally, we emphasize that the analogous PNIPAM‐PHPMA diblock copolymer nanoparticles cannot be synthesized via conventional aqueous PISA because acrylamide‐based precursor blocks exhibit very poor block efficiency when employing methacrylic monomers such as HPMA.

**Figure 4:**
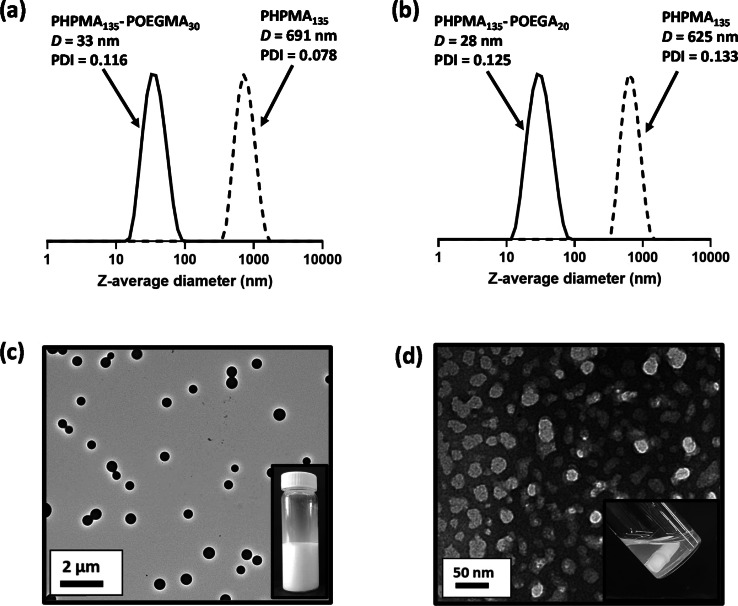
Normalized DLS intensity‐average particle size distributions recorded at 20 °C for 0.1 % w/w aqueous dispersions of (a) charge‐stabilized PHPMA_135_ latex particles and the corresponding sterically‐stabilized PHPMA_135_‐POEGMA_30_ nanoparticles; (b) Charge‐stabilized PHPMA_135_ latex particles and the corresponding sterically‐stabilized PHPMA_135_‐POEGA_20_ nanoparticles. (c) TEM image recorded after drying the PHPMA_135_ latex, which forms a free‐flowing turbid dispersion at 8.0 % w/w solids (see inset digital photograph). (d) TEM image recorded after drying an aqueous dispersion of PHPMA_135_‐POEGMA_30_ nanoparticles. These nanoparticles afford a relatively transparent free‐flowing dispersion at 11 % w/w solids (see inset digital photograph).

As mentioned above, the turbidity of the initial milky‐white PHPMA latex is substantially reduced after chain extension with either OEGMA or OEGA. This suggests a significant reduction in particle size. Accordingly, DLS was used to assess the hydrodynamic diameter of the original PHPMA latex and the final diblock copolymer nanoparticles at 0.1 % w/w solids. A hydrodynamic z‐average diameter of around 600–700 nm was obtained for two examples of PHPMA_135_ latex (Figure 4a and b). However, the corresponding PHPMA_135_‐POEGMA_30_ and PHPMA_135_‐POEGA_20_ nanoparticles exhibited much smaller hydrodynamic diameters of 33 and 28 nm, respectively. This substantial reduction in particle size was confirmed by TEM analysis of each precursor PHPMA_135_ latex and the final PHPMA_135_‐POEGMA_30_ and PHPMA_135_‐POEGA_20_ nanoparticles (Figure 4c, d, and S5a). In contrast, no reduction in turbidity was observed for the attempted synthesis of PHPMA_135_‐PGMA_55_ and PHPMA_135_‐PDMAC_55_ nanoparticles (Figure S6a, b) which is consistent with the uncontrolled homopolymerization of these monomers within the aqueous phase, as discussed above (Figure S4).

No significant reduction in turbidity was also observed for the PHPMA_135_‐PNIPAM_44_ nanoparticles (Figure S6c). However, DLS analysis of these nanoparticles at 20 °C indicated a hydrodynamic z‐average diameter of 111 nm, while TEM studies confirmed the formation of spherical nanoparticles with an estimated number‐average core diameter of 97 nm (based on the analysis of 50 nanoparticles, Figure S5b). Thus, these sterically stabilized PHPMA_135_‐PNIPAM_44_ nanoparticles are significantly larger than the PHPMA_135_‐POEGMA_30_ and PHPMA_135_‐POEGA_20_ nanoparticles, which have z‐average diameters of 33 and 28 nm, respectively (Table S1). Accordingly, this reverse sequence PISA synthesis was repeated, with a PNIPAM DP of 60 being targeted in order to reduce the particle size. Successful chain extension of the PHPMA_135_ precursor was again achieved, as indicated by GPC studies (Figure S4g, h). A hydrodynamic z‐average diameter of 26 nm was indicated by DLS while TEM studies confirmed the formation of spherical nanoparticles with a number‐average core diameter of approximately 18 nm (based on the analysis of 50 nanoparticles; Figure S5c). Moreover, a more significant reduction in dispersion turbidity was also observed, which is consistent with the formation of smaller nanoparticles (Figure S6d).

**Figure 5:**
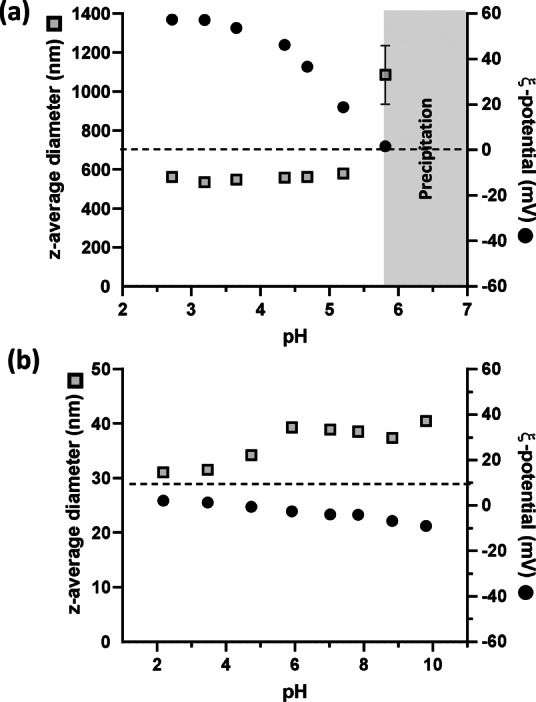
Variation in z‐average diameter and ξ‐potential with pH for (a) charge‐stabilized PHPMA_135_ latex and (b) sterically‐stabilized PHPMA_135_‐POEGMA_30_ nanoparticles [a 0.1 % w/w aqueous dispersion in 1 mM KCl was initially prepared at pH 3 and the solution pH was adjusted using NaOH or HCl].

DLS and aqueous electrophoresis studies were undertaken to assess (i) the colloidal stability of the PHPMA latex over a range of pH and (ii) to investigate any change in electrophoretic behavior after chain extension of this latex when using OEGMA (Figure 5). ξ‐potentials of approximately +40 to +60 mV were observed for the PHPMA particles at pH 2.5–4.5 owing to protonation of the morpholine‐based end‐groups located at the latex surface. Above pH 4.5, these morpholine groups become progressively deprotonated, resulting in loss of surface charge and a concomitant reduction in the ξ‐potential to +19 mV. This is accompanied by a modest increase in the apparent particle diameter from 530 nm at pH 3 up to 580 nm at pH 5.2. At pH 5.8, the ξ‐potential is approximately zero, indicating that the latex particles bear no net surface charge under such conditions. This results in incipient flocculation, as indicated by a dramatic increase in the apparent z‐average diameter up to 1090 nm. Above pH 5.8, macroscopic precipitation of the charge‐stabilized PHPMA latex occurs. In striking contrast, the PHPMA_135_‐POEGMA_30_ nanoparticles remain colloidally stable from pH 2 to pH 10, as indicated by a z‐average diameter of 31–40 nm. Moreover, the ξ‐potential remains relatively constant and reasonably close to zero (from +2 to −9 mV) across this pH range. This is consistent with the majority of the morpholine end‐groups being located within the nanoparticle interior, with the non‐ionic POEGMA_30_ chains acting as a steric stabilizer.

One common feature for the three monomers that enable successful chain extension of the PHPMA latex is that their corresponding homopolymers (i.e. POEGMA, POEGA and PNIPAM) each exhibit inverse temperature solubility behavior^[106]^ and have been employed for the design of thermoresponsive hydrogels.[^107^, ^108^, ^109^, ^110^] In this context, the PHPMA‐PNIPAM formulation is particularly interesting as the reaction temperature (43 °C) is above the lower critical solution temperature (LCST) for PNIPAM homopolymer, which is approximately 32 °C.[^106^, ^111^] Hence the PNIPAM block should be insoluble at the reaction temperature, leading to the formation of relatively large latex particles comprising double‐hydrophobic diblock copolymer chains. To test this hypothesis, a small aliquot of the PHPMA_135_‐PNIPAM_44_ diblock copolymer dispersion was extracted from the hot reaction solution once the NIPAM polymerization was complete. This aliquot was then diluted with a mildly acidic aqueous solution (pH 3) preheated to 45 °C in order to maintain the solution temperature. The resulting 0.1 % w/w copolymer dispersion was then analyzed by DLS at 45 °C. This protocol produced a hydrodynamic z‐average diameter of 644 nm, which is comparable to that observed for the corresponding precursor PHPMA latex (Figure S7 and Table S1). Furthermore, TEM studies confirmed the presence of relatively large particles of comparable size to the original PHPMA_135_ latex (Figure S7). However, cooling the final 9 % w/w PHPMA_135_‐PNIPAM_44_ dispersion to 20 °C followed by dilution led to a substantial reduction in particle size, with DLS studies indicating a hydrodynamic diameter of 111 nm in this case (Figure S7). This suggests that the PNIPAM chains become hydrophilic on cooling below their LCST and hence can act as a steric stabilizer for the hydrophobic PHPMA cores.

**Figure 6:**
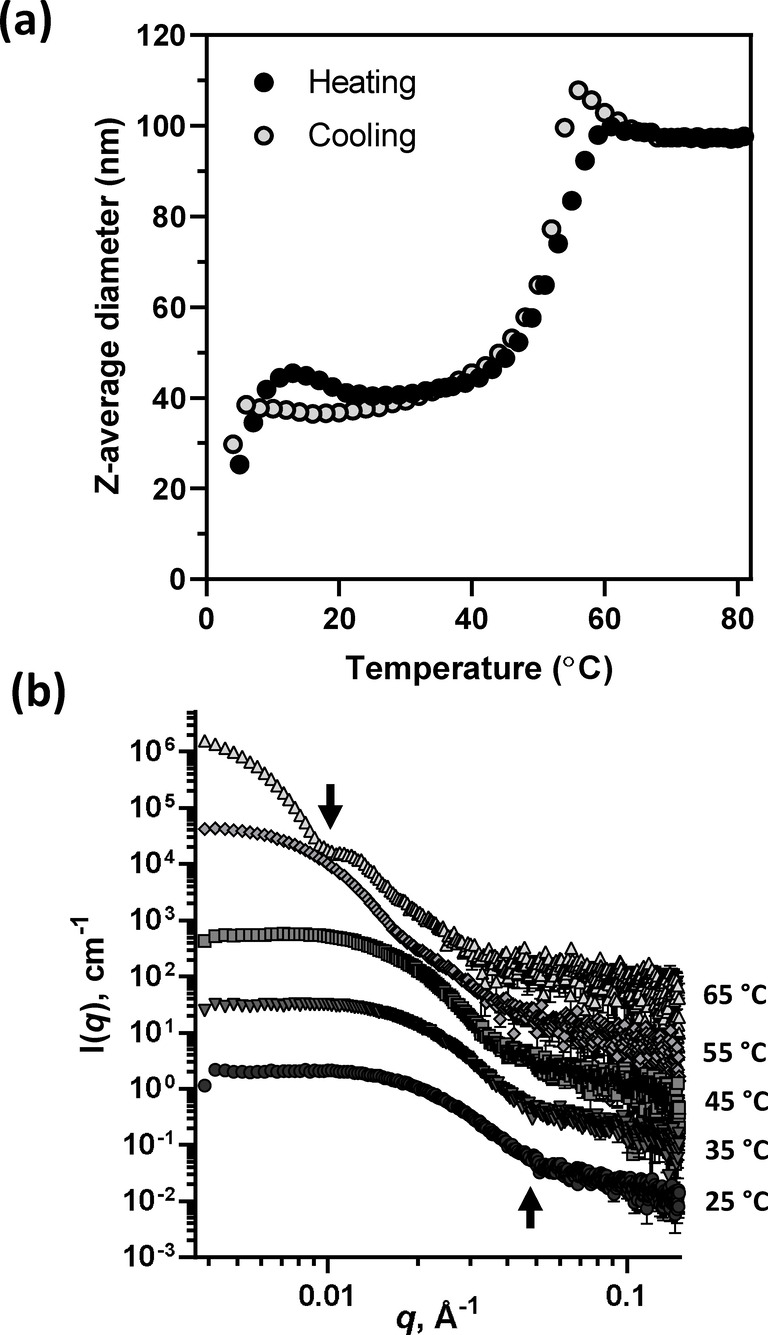
a) Variation in z‐average diameter and polydispersity (PDI) with temperature obtained by dynamic light scattering (DLS) studies of a 0.1 % w/w aqueous dispersion of PHPMA_135_‐POEGMA_30_ nanoparticles. b) SAXS patterns recorded for a 1.0 % w/w aqueous dispersion of PHPMA_135_‐POEGMA_30_ nanoparticles on heating from 25 °C to 65 °C. The progressive shift in intensity minima (indicated on two of the patterns with arrows) to lower *q* indicates a gradual increase in volume‐average diameter at higher temperature.

Amphiphilic PHPMA‐based diblock copolymer nano‐objects are well‐known to be thermoresponsive in the PISA literature.[^72^, ^99^, ^112^] Moreover, POEGMA homopolymer is known to exhibit inverse temperature solubility behavior.^[106]^ Thus, we decided to investigate the thermoresponsive behavior of the PHPMA_135_‐POEGMA_30_ nanoparticles (Figure 6). Between 20 and 40 °C, DLS studies indicate that well‐defined near‐monodisperse spherical nanoparticles are obtained with a z‐average diameter of around 40 nm and a DLS polydispersity of 0.100. The particle size increases significantly on heating above 40 °C, reaching approximately 100 nm diameter at 61 °C. This thermoresponsive behavior proved to be fully reversible: the original particle diameter is regained on cooling to 20 °C. Moreover, the z‐average diameter of the nanoparticles is reduced on cooling below 20 °C with a concomitant reduction in the scattered light intensity, with broader size distributions being obtained under such conditions (Figure 6a). The latter observations suggest that the spherical nanoparticles that are formed at or above ambient temperature undergo disassembly on cooling to subambient temperatures to form weakly interacting copolymer chains. Such thermoresponsive behavior is likely to be related to (i) the temperature‐dependent degree of partial solvation (plasticization) of the PHPMA chains[^31^, ^113^, ^114^, ^115^, ^116^, ^117^] and (ii) the inverse temperature solubility behavior exhibited by the POEGMA stabilizer chains, which become more hydrated at lower temperature.[^106^, ^109^, ^110^]

The temperature‐dependent behavior of these PHPMA_135_‐POEGMA_30_ nanoparticles was further assessed using SAXS (Figure 6b). A zero gradient is observed in the low *q* (Guinier) region of the scattering pattern at 25 °C, which is characteristic of spherical particles. This gradient remains unchanged up to 55 °C, suggesting that the original spherical morphology is retained on heating. Unfortunately, the increase in particle size at higher temperature (e.g. 65 °C) combined with the limited *q* range of our experimental set‐up did not allow the low *q* gradient to be determined under such conditions.

An intensity minimum in the Porod region (high *q*) is observed at all temperatures investigated. On heating from 25 °C to 65 °C, this intensity minimum (see arrows in Figure 6b) shifts to lower *q*, indicating an increase in particle size. An approximate core diameter (*D*_core_) was calculated using the equation *D*_core_=2(4.49/*q*), where the *q* value corresponds to that for the intensity minimum.^[118]^ As expected, the core diameter of the PHPMA_135_‐POEGMA_30_ nanoparticles progressively increases with respect to temperature (from 18 nm at 25 °C up to 90 nm at 65 °C). This suggests a corresponding increase in the mean aggregation number, *N*_agg_, with temperature. Assuming fully dehydrated nanoparticle cores, *N*_agg_ values were estimated from these core diameters using the equation *N*_agg_=*V*_core_/*V*_PHPMA_, where *V*_core_ is the volume of the nanoparticle core and *V*_PHPMA_ is the volume occupied by an individual PHPMA block (Figure S8a). On heating from 25 °C to 65 °C, *N*_agg_ increases significantly from 105 up to 14 100. This suggests that each of the larger nanoparticles comprise approximately 135 of the initial small spherical nanoparticles. Furthermore, the volume‐average core diameter determined by SAXS was always smaller than the corresponding z‐average diameter determined by DLS at any given temperature (Figure S8b). This is because the latter technique reports the overall hydrodynamic diameter, which includes the steric stabilizer chains. Moreover, this difference is reduced at higher temperature, which suggests partial dehydration of the steric stabilizer chains. This is physically reasonable because POEGMA becomes progressively dehydrated at higher temperature prior to its macroscopic precipitation at the cloud point temperature.^[106]^

The thermoresponsive behavior of PHPMA_135_‐POEGA_20_ and PHPMA_135_‐PNIPAM_60_ nanoparticles was also analyzed by DLS (Figure S9). Like the PHPMA_135_‐POEGMA_30_ system, the PHPMA_135_‐POEGA_20_ and PHPMA_135_‐PNIPAM_60_ nanoparticles each form larger particles at higher temperatures. In the case of PHPMA_135_‐POEGA_20_, a substantial increase in z‐average diameter is observed at around 65 °C, and particles of 238 nm diameter are formed at 81 °C. However, this change in size proved to be irreversible on cooling, at least for the 0.1 % w/w copolymer dispersion required for DLS studies (Figure S9a). In contrast, the 25 nm PHPMA_135_‐PNIPAM_60_ nanoparticles exhibited reasonably good thermoreversibility, with 60 nm diameter nanoparticles being formed on heating to 81 °C and 25 nm diameter nanoparticles observed on returning to 20 °C.

## Conclusion

We report a new paradigm‐breaking surfactant‐free aqueous PISA formulation that enables the structure‐directing hydrophobic block to be prepared first when targeting amphiphilic diblock copolymers. This counter‐intuitive reverse sequence approach requires an ionic RAFT agent to confer charge stabilization on the precursor latex, which is prepared via RAFT aqueous dispersion polymerization of 2‐hydroxypropyl methacrylate (HPMA). This hydrophobic precursor can be chain‐extended with methacrylic (OEGMA), acrylic (OEGA), or acrylamide (NIPAM) comonomers to produce a range of diblock copolymer nanoparticles via a highly efficient wholly aqueous one‐pot formulation. Importantly, both PHPMA‐POEGA and PHPMA‐PNIPAM copolymers cannot be synthesized using conventional PISA. For such reverse sequence PISA formulations, the second monomer (e.g. OEGMA, OEGA or NIPAM) diffuses into the hydrophobic PHPMA latex particles, which act as the locus for the second‐stage polymerization. In contrast, certain other water‐soluble monomers (e.g. GMA or DMAC) prefer to remain within the aqueous continuous phase, rather than migrating into the PHPMA particles. This alternative scenario leads to homopolymerization via uncontrolled free radical polymerization, as confirmed by UV GPC analysis. For the reverse sequence PISA syntheses reported herein, the precursor charge‐stabilized PHPMA latex particles are relatively large at around 600 nm diameter, whereas the final sterically stabilized diblock copolymer nanoparticles are as small as 30–40 nm diameter. Thus, a remarkable change in morphology occurs during the synthesis of the hydrophilic steric stabilizer chains. Finally, PHPMA_135_‐POEGMA_30_ exhibited reversible thermoresponsive behavior: it forms relatively small nanoparticles of 33 nm diameter at 20 °C but significantly larger particles on heating to 60–65 °C. Given the excellent control over the molecular weight distribution and minimal levels of residual monomer, we envisage that this new reverse sequence PISA route will be a useful addition to the synthetic polymer chemist's toolbox for the rational synthesis of functional block copolymer nanoparticles, particularly for formulations that are not feasible by conventional aqueous PISA.

## Conflict of interest

The authors declare no conflict of interest.

1

## Supporting information

As a service to our authors and readers, this journal provides supporting information supplied by the authors. Such materials are peer reviewed and may be re‐organized for online delivery, but are not copy‐edited or typeset. Technical support issues arising from supporting information (other than missing files) should be addressed to the authors.

Supporting Information

## Data Availability

The data that support the findings of this study are available in the Supporting Information of this article.
