# Supplementary material for: Reverse Sequence Polymerization‐Induced Self‐Assembly in Aqueous Media
Source: Angew Chem Int Ed Engl. 2022 Jul 6;61(33):e202207376. doi: 10.1002/anie.202207376 (PMC9541501; doi:10.1002/anie.202207376)
Supplement: Supplementary file 1 — Supporting Information [file ANIE-61-0-s001.pdf]

Angewandte Chemie
Eine Zeitschrift der Gesellschaft Deutscher Chemiker

Supporting Information

Reverse Sequence Polymerization-Induced Self-Assembly in Aqueous Media

T. J. Neal, N. J. W. Penfold, S. P. Armes**

Experimental **2**
Materials. 2
Characterization Techniques *2*
[1]H Nuclear Magnetic Resonance (NMR) Spectroscopy 2
Gel Permeation Chromatography (GPC) 2
Dynamic Light Scattering (DLS) and Aqueous Electrophoresis 3
Transmission Electron Microscopy (TEM) 3
Small-Angle X-ray Scattering (SAXS) 3
Synthesis Protocols *4*
One-pot reverse sequence PISA synthesis of $PHPMA_{135}$-$POEGMA_{30}$ diblock copolymer nano-objects via RAFT aqueous dispersion polymerization 4
One-pot reverse sequence PISA synthesis of $PHPMA_{135}$-$POEGA_{20}$ diblock copolymer nano-objects via RAFT aqueous dispersion polymerization 5
One-pot reverse sequence PISA synthesis of $PHPMA_{135}$-$PNIPAM_{x}$ diblock copolymer nano-objects via RAFT aqueous dispersion polymerization 5
Attempted one-pot reverse sequence PISA synthesis of $PHPMA_{135}$-$PGMA_{55}$ diblock copolymer nano-objects via RAFT aqueous dispersion polymerization 6
Attempted one-pot reverse sequence PISA synthesis of $PHPMA_{135}$-$PDMAC_{55}$ diblock copolymer nano-objects via RAFT aqueous dispersion polymerization 6
Supporting figures and table **8**
References **14**

Experimental

Materials. The morpholine-functionalized trithiocarbonate-based RAFT agent (denoted MPETTC) used in this study was prepared as described in the literature.[1] 2-Hydroxypropyl methacrylate (HPMA; 97%) and glycerol monomethacrylate (GMA; >99%) were kindly donated by GEO Specialty Chemicals (Hythe, UK). *N*-Isopropylacrylamide (NIPAM) was purchased from Fluorochem (UK), recrystallized from *n*-hexane and dried before use. Oligo(ethylene glycol) methyl ether methacrylate (OEGMA, mean degree of polymerization = 4-5; M_n ~ 300 g mol^{-1}, 99%), oligo(ethylene glycol) methyl ether acrylate (OEGA, mean degree of polymerization = 7-8; M_n ~ 480 g mol^{-1}, 99%), *N,N'*-dimethylacrylamide (DMAC, 99%), sodium benzenesulfonate (97%), deuterium chloride (DCl, 35 % w/w) and deuterium oxide (D_2O, 99%) were purchased from Sigma Aldrich (UK). Deuterated methanol (CD_3OD; 99.8%) was purchased from Cambridge Isotopes Laboratories Ltd (UK). All other solvents were purchased from either VWR International (UK) or Sigma Aldrich (UK) and were HPLC-grade quality. Deionized water was obtained from an Elgastat Option 3A water purification unit with a resistivity of 15 MΩ cm.

Characterization Techniques

^{1}H Nuclear Magnetic Resonance (NMR) Spectroscopy

All ^{1}H NMR spectra were recorded in CD_3OD or D_2O using a 400 MHz Bruker Avance-400 spectrometer operating at 298 K with 16 scans being averaged per spectrum. When investigating the diffusion of water-soluble monomers into the PHPMA latex particles, the latex pH (in H_2O) was adjusted to pH 3 using HCl. Alternatively, the latex was diluted using D_2O (adjusted to pD 3 using DCl) to achieve a 90:10 v/v H_2O/D_2O ratio.

Gel Permeation Chromatography (GPC)

Aqueous homopolymer or copolymer dispersions were diluted to 0.50% w/w using HPLC-grade DMF eluent containing 10 mM LiBr. GPC studies were conducted at 60 °C using a flow rate of 1.0 mL min^{-1} and DMSO (1.0 % v/v) as a flow rate marker. The GPC set-up comprised an Agilent 1260 Infinity series degasser and pump, an

Agilent PL-gel guard column, two Agilent PL-gel Mixed-C columns, a refractive index detector and a UV detector set at a wavelength of 305 nm. Eleven near-monodisperse poly(methyl methacrylate) standards with M_p values ranging from 2,380 g mol^{-1} to 2,200,000 g mol^{-1} were used for calibration.

Dynamic Light Scattering (DLS) and Aqueous Electrophoresis

DLS and aqueous electrophoresis measurements were conducted at 25 °C using a Malvern Instruments Zetasizer Nano ZS series instrument equipped with a 4 mW He−Ne laser (λ = 633 nm) and an avalanche photodiode detector. Scattered light was detected at 173°. Aqueous dispersions were diluted to a final concentration of 0.1% w/w solids using an acidic aqueous solution of 1 mM KCl (adjusted to pH 3 using HCl). Hydrodynamic z-average diameters were calculated via the Stokes-Einstein equation. Zeta (ξ) potentials were calculated using the Henry equation via the Smoluchowski approximation. The aqueous dispersion pH was adjusted using either 0.1-1.0 M HCl or 0.1-1.0 M NaOH as required.

Transmission Electron Microscopy (TEM)

Copper/palladium grids were coated with a thin film of amorphous carbon and then plasma glow-discharged for 20 seconds to afford a hydrophilic surface. A 10 μL droplet of the freshly-prepared 0.1% w/v aqueous copolymer dispersion was placed on the hydrophilic grid for 15 seconds, blotted to remove excess sample and then negatively-stained with uranyl formate solution (0.75% w/v; 10 μL) for a further 15 seconds. Excess stain was removed by blotting and each grid was carefully dried with a vacuum house. TEM studies were performed using a FEI Tecnai Spirit 2 microscope equipped with an Orius SC1000B camera operating at 80 kV.

Small-Angle X-ray Scattering (SAXS)

SAXS patterns were collected using a Xenocs Xeuss 2.0 laboratory beamline equipped with a 2D Dectris Pilatus 1M detector and an Excillum liquid gallium MetalJet X-ray source (λ = 1.34 Å). Aqueous copolymer dispersions were placed in a capillary (2.0 mm diameter) and the sample temperature was controlled using a HFSX350-CAP heating/cooling capillary holding stage (Linkam Scientific Instruments Ltd, Tadworth,

UK). SAXS data were recorded over a scattering vector range of 0.004 Å^{-1} < q < 0.400 Å^{-1}, where $q = \frac{4\pi}{\lambda}\sin\theta$ and θ is one-half of the scattering angle. One-dimensional (1D) scattering curves were obtained by azimuthal binning and averaging of the corresponding two-dimensional (2D) scattering patterns using software packages supplied with the SAXS instruments. Calibration, background subtraction, normalization and further analysis of the 1D data were performed using Irena SAS macros for Igor Pro.[2]

Synthesis Protocols

One-pot reverse sequence PISA synthesis of $PHPMA_{135}$-$POEGMA_{30}$ diblock copolymer nano-objects via RAFT aqueous dispersion polymerization

A 14 mL glass vial was charged with HPMA (0.200 g, 1.38 mmol, MPETTC (4.6 mg, 10.3 µmol; target degree of polymerization, DP = 135) and a magnetic flea. Once the MPETTC had fully dissolved in the HPMA monomer, deionized water (1.36 g) was added and the resulting aqueous solution was adjusted to pH 3 using 0.1 M HCl to afford a yellow solution. A 1.0 mL aqueous solution of VA-44 initiator (0.70 mg, 2.06 µmol; [MPETTC]/[VA-44] molar ratio = 5.0; adjusted to pH 3) was added to the reaction solution. The vial was sealed with a rubber septum and the reaction mixture was sparged with N_2 gas for 15 min. Then the vial was placed in a pre-heated oil bath set at 43 °C. The initial pale-yellow solution became increasingly turbid, eventually affording a milky-white latex dispersion. Meanwhile, OEGMA was sparged with N_2 for 15 min in a separate vial. After the HPMA polymerization had been allowed to proceed for 3 h (>99% HPMA conversion by ^{1}H NMR spectroscopy; 8% w/w solids; DLS diameter = 691 nm; DLS polydispersity = 0.078), degassed OEGMA (0.090 g, 0.300 mmol; target DP = 30) was added to the $PHPMA_{135}$ latex. The OEGMA polymerization was conducted at 43 °C for 16 h and then quenched by exposing the reaction mixture to air while cooling the vial to 20 °C.

One-pot reverse sequence PISA synthesis of $PHPMA_{135}$-$POEGA_{20}$ diblock copolymer nano-objects via RAFT aqueous dispersion polymerization

A 14 mL glass vial was charged with HPMA (0.200 g, 1.38 mmol, MPETTC (4.6 mg, 10.3 µmol; [target DP = 135) and a magnetic flea. Once the MPETTC had fully dissolved in the HPMA monomer, deionized water (1.36 g) was added and the resulting aqueous solution was adjusted to pH 3 using 0.1 M HCl to afford a yellow solution. A 1.0 mL aqueous solution of VA-44 initiator (0.70 mg, 2.06 µmol; [MPETTC]/[VA-44] molar ratio = 5.0; adjusted to pH 3) was added to the reaction solution. The vial was sealed with a rubber septum and the reaction mixture was sparged with N_2 gas for 15 min. Then the vial was placed in a pre-heated oil bath set at 43 °C. The initial pale-yellow solution became increasingly turbid, eventually affording a milky-white latex dispersion. Meanwhile, OEGA was sparged with N_2 for 15 min in a separate vial. After the HPMA polymerization had proceeded for 3 h (>99% HPMA conversion by ^{1}H NMR spectroscopy; 8% w/w solids; DLS diameter = 625 nm; DLS polydispersity = 0.133), degassed OEGA (0.096 g, 0.200 mmol; target DP = 20) was added to the $PHPMA_{135}$ latex. The OEGA polymerization was allowed to proceed for 16 h at 43 °C and then quenched by exposing the reaction mixture to air while cooling the vial to 20 °C.

One-pot reverse sequence PISA synthesis of $PHPMA_{135}$-$PNIPAM_x$ diblock copolymer nano-objects via RAFT aqueous dispersion polymerization

The synthesis of $PHPMA_{135}$-$PNIPAM_{60}$ is shown here as an example. A 14 mL glass vial was charged with HPMA (0.200 g, 1.38 mmol, MPETTC (4.6 mg, 10.3 µmol; target DP = 135) and a magnetic flea. Once the MPETTC had fully dissolved in the HPMA monomer, deionized water (1.36 g) was added and the resulting aqueous solution was adjusted to pH 3 using 0.1 M HCl to afford a yellow solution. A 1.0 mL aqueous solution of VA-44 initiator (0.70 mg, 2.06 µmol; [MPETTC]/[VA-44] molar ratio = 5.0; adjusted to pH 3) was added to the reaction solution. The vial was sealed with a rubber septum and the reaction mixture was sparged with N_2 gas for 15 min. Then the vial was placed in a pre-heated oil bath set at 43 °C. The initial pale-yellow solution became increasingly turbid, eventually affording a milky-white latex dispersion. Meanwhile, NIPAM was dissolved in pH 3 water in a separate vial to afford

a 230 g dm^{-3} aqueous solution, which was sparged with N_2 for 15 min. After the HPMA polymerization had proceeded for 3 h (HPMA conversion = > 99% by ^{1}H NMR spectroscopy; 8% w/w solids; DLS diameter = 576 nm; DLS polydispersity = 0.100), the degassed aqueous NIPAM solution (0.300 mL, 0.200 mmol of NIPAM; target DP = 60) was added to the $PHPMA_{135}$ latex. The NIPAM polymerization was conducted at 43 °C for 16 h and then quenched by exposing the reaction mixture to air while cooling the vial to 20 °C.

Attempted one-pot reverse sequence PISA synthesis of $PHPMA_{135}$-$PGMA_{55}$ diblock copolymer nano-objects via RAFT aqueous dispersion polymerization

A 14 mL glass vial was charged with HPMA (0.300 g, 2.08 mmol, MPETTC (7.0 mg, 15.4 µmol; target DP = 135) and a magnetic flea. Once the MPETTC had fully dissolved in the HPMA monomer, deionized water (2.54 g) was added, and the resulting aqueous solution was adjusted to pH 3 using 0.1 M HCl to afford a yellow solution. A 1.0 mL aqueous solution of VA-44 initiator (1.0 mg, 3.08 µmol; [MPETTC]/[VA-44] molar ratio = 5.0; adjusted to pH 3) was added to the reaction solution. The vial was sealed with a rubber septum and the reaction mixture was sparged with N_2 gas for 15 min. Then the vial was placed in a pre-heated oil bath set at 43 °C. The initial pale-yellow solution became increasingly turbid, eventually affording a milky-white latex dispersion. Meanwhile, GMA monomer was sparged with N_2 for 15 min. After allowing the HPMA polymerization to proceed for 3 h (>99% HPMA conversion by ^{1}H NMR spectroscopy; 8% w/w solids; DLS diameter = 664 nm; DLS polydispersity = 0.184), degassed GMA (0.13 g, 0.829 mmol, target DP = 55) was added to the $PHPMA_{135}$ latex. The GMA polymerization was conducted at 43 °C for 16 h and then quenched by exposing the reaction mixture to air while cooling the vial to 20 °C.

Attempted one-pot reverse sequence PISA synthesis of $PHPMA_{135}$-$PDMAC_{55}$ diblock copolymer nano-objects via RAFT aqueous dispersion polymerization

A 14 mL glass vial was charged with HPMA (0.200 g, 1.39 mmol, MPETTC (4.6 mg, 10.3 µmol; target DP = 135) and a magnetic flea. Once the MPETTC had fully dissolved in the HPMA monomer, deionized water (1.36 g) was added, and the resulting aqueous solution was adjusted to pH 3 using 0.1 M HCl to afford a yellow solution. A

1.0 mL aqueous solution of VA-44 initiator (0.7 mg, 2.06 µmol; [MPETTC]/[VA-44] molar ratio = 5.0; adjusted to pH 3) was added to the reaction solution. The vial was sealed with a rubber septum and the reaction mixture was sparged with N_2 gas for 15 min. Then the vial was placed in a pre-heated oil bath set at 43 °C. The initial pale-yellow solution became increasingly turbid, eventually affording a milky-white latex dispersion. Meanwhile, DMAC monomer was sparged with N_2 for 15 min. After allowing the HPMA polymerization to proceed for 3 h (>99% HPMA conversion by ^{1}H NMR spectroscopy; 8% w/w solids; DLS diameter = 590 nm; DLS polydispersity = 0.071), degassed DMAC (0.055 g, 0.552 mmol, target DP = 55) was added to the $PHPMA_{135}$ latex. The DMAC polymerization was conducted at 43 °C for 16 h and then quenched by exposing the reaction mixture to air while cooling the vial to 20 °C.

Supporting figures and table

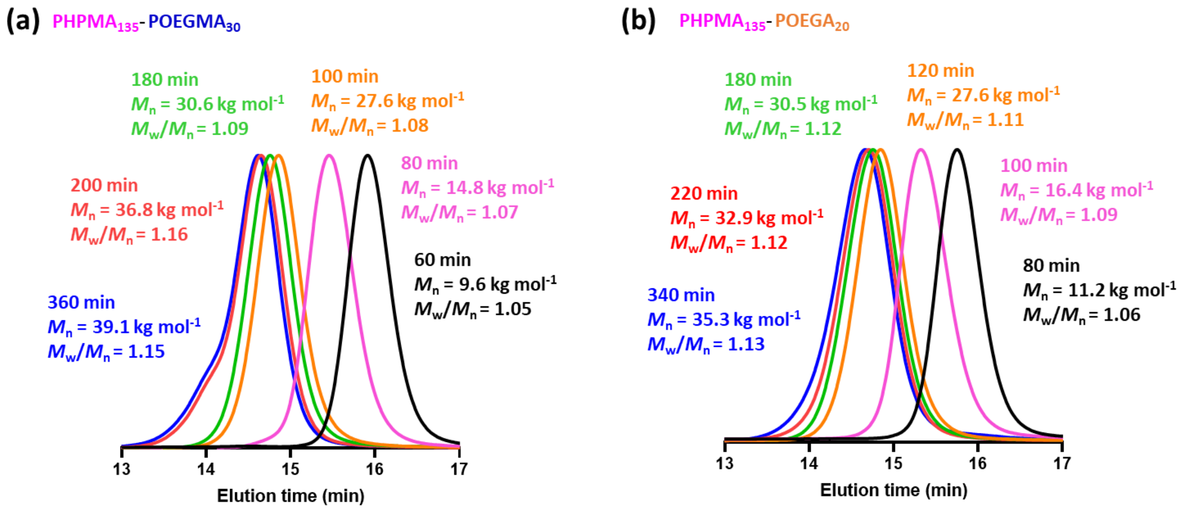

Figure S1. (a) DMF GPC curves recorded during the reverse sequence aqueous PISA synthesis of PHPMA135-POEGMA30 nanoparticles. (b) DMF GPC curves recorded during the reverse sequence aqueous PISA synthesis of PHPMA135-POEGA20 nanoparticles.

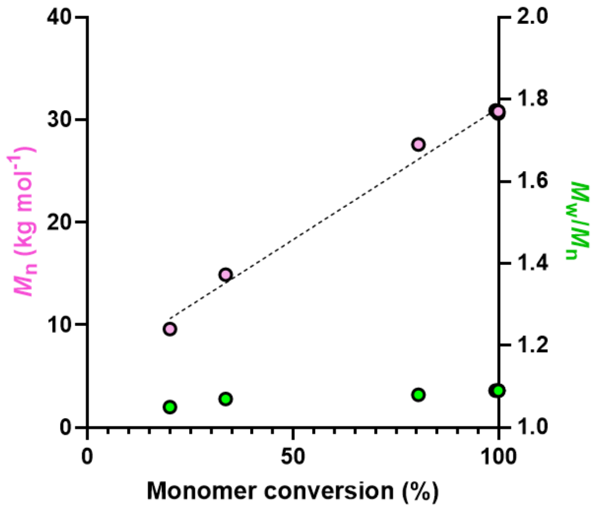

Figure S2. Evolution in number-average molecular weight (M_n) and dispersity (M_w/M_n) with monomer conversion observed during the RAFT aqueous dispersion polymerization of HPMA using an MPETTC RAFT agent and a VA-44 azo initiator. Polymerization conditions: [HPMA]/[MPETTC] molar ratio = 135, [MPETTC]/[VA-44] molar ratio = 5.0, targeting 8% w/w solids at pH 3.

Table S1. Summary of the target composition, z-average particle diameter (and DLS polydispersity) for (i) six precursor PHPMA latexes and (ii) the corresponding final diblock copolymer nanoparticles obtained when using OEGMA, OEGA or NIPAM.

Sample	PHPMA precursor DP	PHPMA latex z-average diameter (nm)	Comonomer used for second block	Target DP for second block	Diblock copolymer nanoparticle z-average diameter (nm)
1	135	691 (0.078)	OEGMA	30	33 (0.116)
2	135	625 (0.133)	OEGA	20	28 (0.125)
3	135	664 (0.184)	GMA	55	n.d.
4	135	590 (0.071)	DMAC	55	n.d.
5	135	574 (0.100)	NIPAM	60	26 (0.142)
6	135	644 (0.084)	NIPAM	44	111 (0.073)

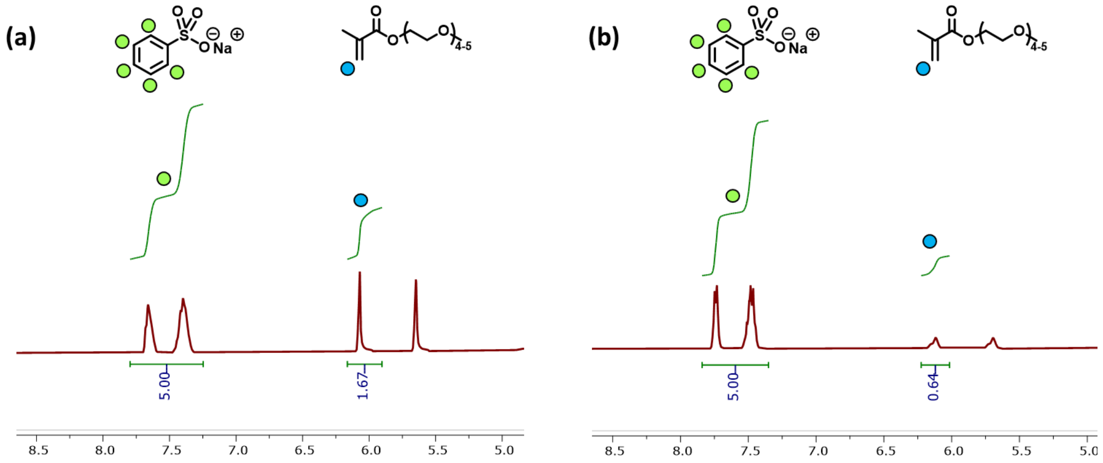

Figure S3. ^{1}H NMR spectra recorded using 50 g dm^{-3} sodium benzenesulfonate as an external standard for (a) OEGMA monomer (72 mg) dissolved in a binary mixture comprising 2.00 mL acidified H_2O (adjusted to pH 3 using HCl) plus 0.20 mL D_2O and (b) OEGMA monomer (72 mg) dissolved in a binary mixture comprising 2.00 mL of an 8 % w/w aqueous dispersion of PHPMA$_{135}$ latex particles plus 0.20 mL D_2O. The OEGMA proton signals become significantly attenuated in the presence of the PHPMA latex, suggesting that some of this monomer preferentially migrates within these particles rather than remaining in the aqueous phase.

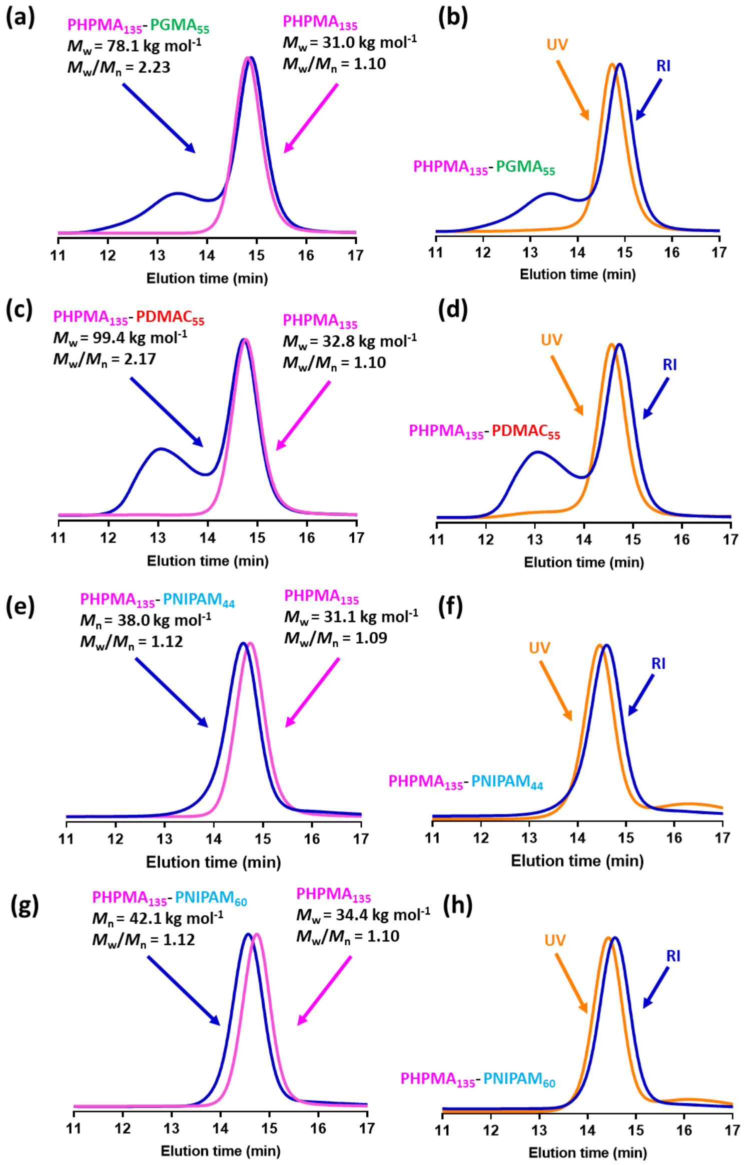

Figure S4. (a) DMF GPC curves recorded for the $PHPMA_{135}$ latex precursor and the corresponding $PHPMA_{135}$-$PGMA_{55}$ nanoparticles using a refractive index detector. (b) Comparison of GPC curves recorded using a refractive index and a UV detector (λ = 305 nm) for these two samples. (c) DMF GPC curves recorded for the $PHPMA_{135}$ latex precursor and the corresponding $PHPMA_{135}$-$PDMAC_{50}$ nanoparticles using a refractive index detector. (d) Comparison of GPC curves recorded using a refractive index and a UV detector (λ = 305 nm) for these two samples. (e) DMF GPC curves recorded for the $PHPMA_{135}$ latex precursor and the corresponding $PHPMA_{135}$-$PNIPAM_{44}$ nanoparticles using a refractive index detector. (f) Comparison of GPC curves recorded using a refractive index and a UV detector (λ = 305 nm) for these two samples. (g) DMF GPC curves recorded for the $PHPMA_{135}$ latex precursor and the corresponding $PHPMA_{135}$-$PNIPAM_{60}$ nanoparticles using a refractive index detector. (h) Comparison of GPC curves recorded using a refractive index and a UV detector (λ = 305 nm) for these two samples.

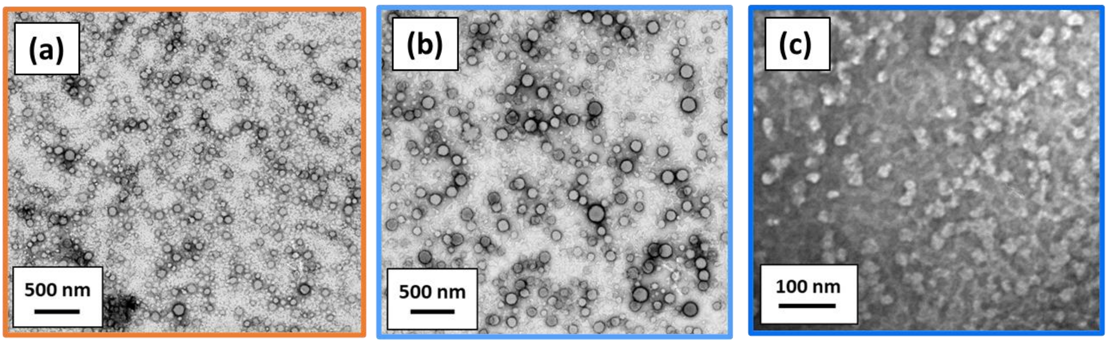

Figure S5. TEM images recorded after drying the following dilute aqueous diblock copolymer dispersions: (a) $PHPMA_{135}$-$POEGA_{20}$ nanoparticles, (b) $PHPMA_{135}$-$PNIPAM_{44}$ nanoparticles and (c) $PHPMA_{135}$-$PNIPAM_{60}$ nanoparticles.

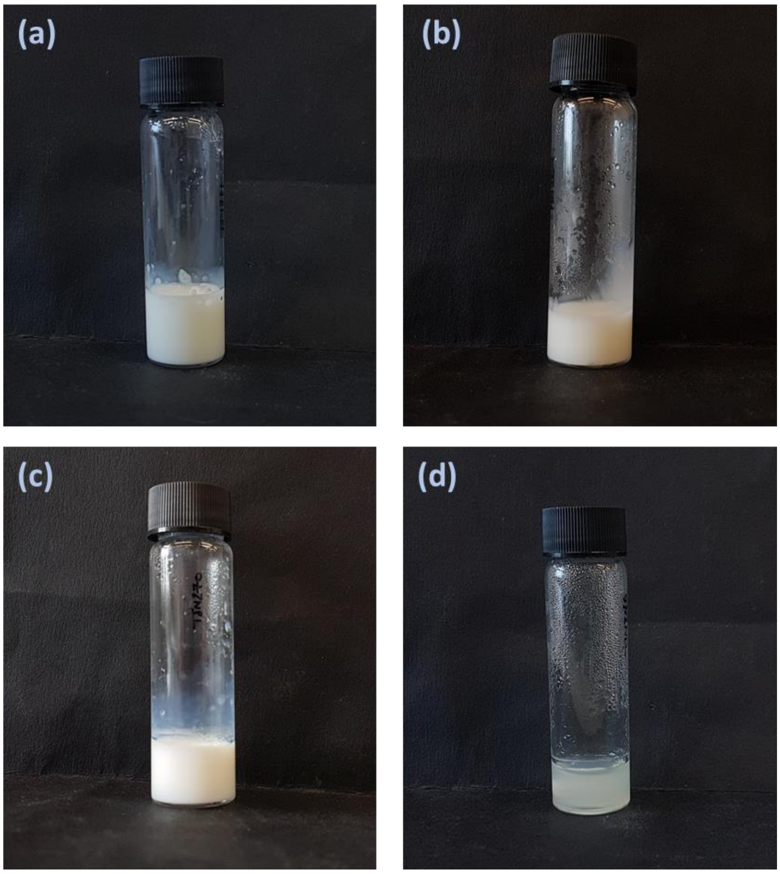

Figure S6. Digital photographs recorded for 11% w/w aqueous dispersions obtained after targeting the following diblock copolymer nanoparticles: (a) $PHPMA_{135}$-$PGMA_{55}$ nanoparticles, (b) $PHPMA_{135}$-$PDMAC_{55}$ (c) $PHPMA_{135}$-$PNIPAM_{44}$ and (d) $PHPMA_{135}$-$PNIPAM_{60}$ nanoparticles. [N.B. For the first two examples, attempted chain extension was unsuccessful: instead, the GMA/DMAC monomer was simply homopolymerized within the aqueous phase].

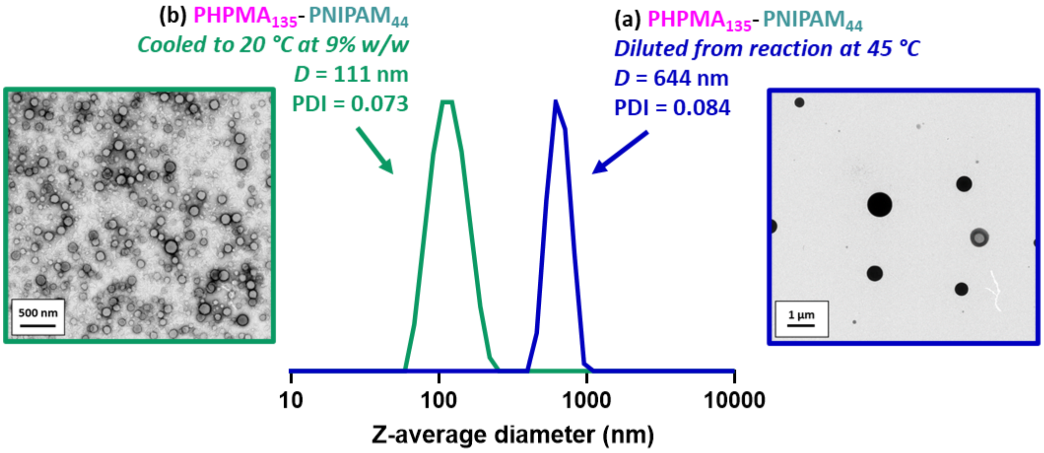

Figure S7. Normalized DLS intensity-average particle size distributions recorded for a 0.1% w/w aqueous dispersion of $PHPMA_{135}$-$PNIPAM_{44}$ particles: (a) on dilution of the hot reaction solution using an acidic aqueous solution (pH 3) at a constant temperature of 45 °C (see right-hand TEM image) or (b) when the same 9% w/w dispersion was first cooled to 20 °C prior to dilution to 0.1% w/w (see left-hand TEM image).

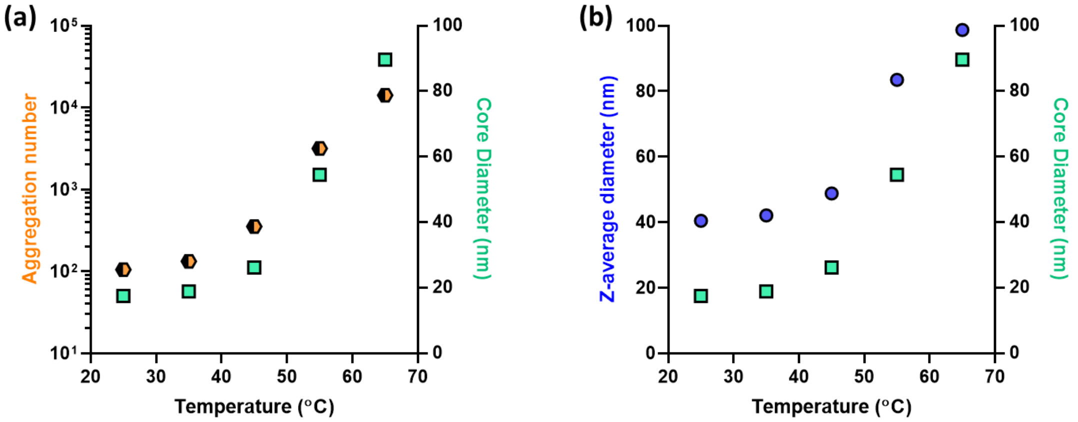

Figure S8. (a) Temperature dependence of the aggregation number and core diameter for the $PHPMA_{135}$-$POEGMA_{30}$ particles as determined by SAXS. (b) Comparison between the z-average diameter and the core diameter as a function of temperature as determined by DLS and SAXS, respectively.

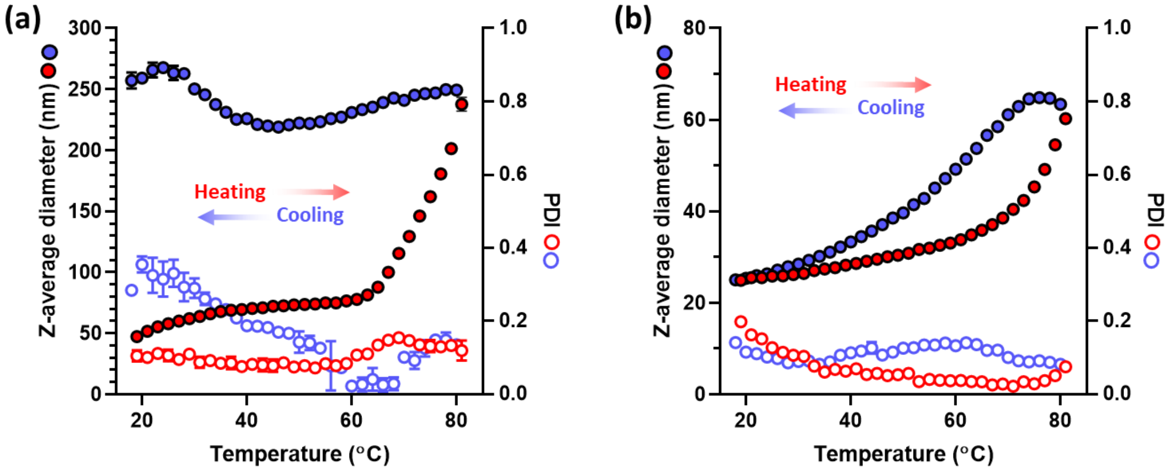

Figure S9. Variation in z-average diameter and polydispersity (PDI) with temperature obtained by dynamic light scattering (DLS) studies of 0.1% w/w aqueous dispersions of (a) $PHPMA_{135}$-$POEGA_{20}$ and (b) $PHPMA_{135}$-$PNIPAM_{60}$ nanoparticles.

References

(1) Penfold, N. J. W.; Lovett, J. R.; Warren, N. J.; Verstraete, P.; Smets, J.; Armes, S. P. PH-Responsive Non-Ionic Diblock Copolymers: Protonation of a Morpholine End-Group Induces an Order-Order Transition. *Polym. Chem.* **2016**, *7*, 79–88. https://doi.org/10.1039/C5PY01510C.

(2) Ilavsky, J.; Jemian, P. R. Irena : Tool Suite for Modeling and Analysis of Small-Angle Scattering . *J. Appl. Crystallogr.* **2009**, *42* (2), 347–353. https://doi.org/10.1107/s0021889809002222.
